# Modulation of group B *Streptococcus* infection and vaginal cell inflammatory signaling *in vitro* by *Lactobacillus crispatus*-loaded electrospun fibers

**DOI:** 10.1128/iai.00170-25

**Published:** 2025-08-27

**Authors:** Nagwa El-Baz, Anthony Kyser, Mohamed Y. Mahmoud, Christopher Z. Farrell, Sierra Ginocchio, Hermann B. Frieboes, Ryan S. Doster

**Affiliations:** 1 Department of Medicine, Division of Infectious Diseases, University of Louisville School of Medicine 12254, Louisville, Kentucky, USA; 2 Department of Bioengineering, University of Louisville Speed School of Engineering 223102, Louisville, Kentucky, USA; 3 Department of Toxicology and Forensic Medicine, Faculty of Veterinary Medicine, Cairo University 63526, Giza, Egypt; 4 Department of Microbiology and Immunology, University of Louisville School of Medicine 12254, Louisville, Kentucky, USA; 5 Department of Pharmacology and Toxicology, University of Louisville 536767, Louisville, Kentucky, USA; 6 Center for Predictive Medicine, University of Louisville 5170, Louisville, Kentucky, USA; University of California Davis, Davis, California, USA

**Keywords:** women's health, electrospun fibers, Group B* Streptococcus*, *Lactobacillus*

## Abstract

Vaginal colonization by *Streptococcus agalactiae,* also known as Group B *Streptococcus* (GBS), is a major risk factor for ascending infections, preterm birth, and neonatal sepsis. Current GBS prevention efforts include routine GBS perinatal screening and intrapartum antibiotic prophylaxis, which decrease the rate of early-onset neonatal sepsis, but have drawbacks that include impacting the infant’s developing microbiome. *Lactobacillus*-dominant vaginal microbiomes provide protection against pathogens such as GBS, and using probiotics as an antibiotic-free approach to limit GBS colonization is of increasing interest. In this study, we investigated the ability of *Lactobacillus crispatus-*loaded electrospun fibers to deliver live *L. crispatus* cells in an *in vitro* vaginal epithelial cell model, modulate GBS infection establishment and persistence, and alter vaginal cell inflammatory signaling. Our data demonstrate that electrospun fibers deliver viable *L. crispatus* to the surface of vaginal epithelial cells and that *L. crispatus* modulates vaginal cell inflammatory signaling by decreasing inflammatory IL-8 release and increasing anti-inflammatory IL-1RA secretion during established GBS infection. Treatment of pre-established GBS infection with electrospun fibers with or without *L. crispatus* decreased GBS burden at 24 hours, suggesting *L. crispatus*-dependent and -independent anti-GBS activity, and *L. crispatus* elicited an anti-inflammatory response via IL-1RA release. Overall, the data highlight the potential of electrospun fibers as a feasible probiotic delivery platform with antibacterial activity against GBS and which provides commensal lactobacilli capable of modulating host-pathogen interactions and inflammatory signaling of the vaginal epithelium.

## INTRODUCTION

The vaginal microbiome has significant impacts on female sexual and reproductive health. Early studies of the vaginal microbiome demonstrated clustering of microbial communities into five major community state types (CSTs), four of which are dominated by different *Lactobacillus* species and one (CST IV) represented by a diverse community associated with increased abundance of anaerobic bacteria and higher vaginal pH (1). *Lactobacillus*-dominant CSTs promote urogenital health by lowering vaginal pH (2), reducing colonization by potential pathogens (3), and minimizing vaginal inflammation through immunomodulatory effects (4). Non-*Lactobacillus* dominant vaginal microbiotas are associated with several disease states including urinary tract infections (5), preterm birth (6), miscarriage (7), sexually transmitted infections (8–10), pelvic inflammatory diseases (11), and cervical cancer (12). Given the consequences of a non-*Lactobacillus* dominant vaginal flora, particularly during years of reproductive potential, a pressing need exists to investigate strategies to increase beneficial bacteria and stabilize a healthy vaginal microbiome.

*Streptococcus agalactiae,* also known as Group B *Streptococcus* (GBS), is an opportunistic pathogen that colonizes the gastrointestinal and lower female reproductive tracts of 15%–30% of healthy individuals (13, 14). During pregnancy, rectovaginal GBS colonization is a major risk factor for invasive GBS disease as GBS can ascend through the urogenital tract into the female reproductive tract and infect the fetal membranes, causing pregnancy complications such as chorioamnionitis, preterm premature rupture of membranes, and stillbirth (15–17). GBS can be transmitted *in utero* to the neonate or during delivery through inhalation of contaminated vaginal fluid, leading to pneumonia, meningitis, and sepsis (18, 19). Neonatal GBS disease is classified into early-onset disease (EOD), which occurs during the first week of life, or late-onset disease (LOD), occurring after the first week through the third month of life (19). In the United States, the Centers for Disease Control and Prevention recommends that all pregnant women undergo perinatal GBS screening in the third trimester and administration of intrapartum antibiotic prophylaxis (IAP) during delivery for those who test positive (20). Administration of IAP to GBS-positive mothers decreases rates of EOD by over 80% but does not affect rates of LOD (21), but increasing concerns exist regarding IAP’s impact on perturbations of the neonate’s developing gut microbiota, emergence of antimicrobial-resistant organisms, and long-term adverse effects on child immunity and behavior (22–26). Given the number of women and neonates exposed to antibiotics and concern for the long-term effects of IAP, new strategies are needed to limit GBS rectovaginal colonization during pregnancy.

Using constituents of the microbiome to limit niches for potential pathogens like GBS is of increasing interest. In studies examining the vaginal microbiota from pregnant women, *L. crispatus* abundance is negatively associated with GBS colonization, suggesting a protective role of this *Lactobacillus* species (27). Use of *Lactobacillus* probiotics to limit GBS rectovaginal colonization has been investigated but has yielded mixed results, possibly related to differences in routes of administration (oral or intravaginal), the particular *Lactobacillus* species or isolate used, and discrepancies in dosing (frequency and amount) (28–30). To date, the U.S. Food and Drug Administration has not authorized use of any probiotic therapies for prevention or treatment of vaginal infections.

Lack of success with traditional vaginal and oral delivery mechanisms has prompted new approaches to deliver probiotic bacteria. Electrospun nanofibers (EFs) composed of biocompatible polymers have emerged as a promising alternative for local therapeutic delivery, as recently reviewed (31), seeking to tackle vaginal infections and microbiome disturbances (32–35). Mucoadhesive polymers such as polyethylene oxide (PEO) in the form of fibers have demonstrated adherence to human vaginal tissue and probiotic eluting properties at vaginal pH (36–38). Polymers with different bond strengths modify the release profile of drug or living agents depending on their polymeric degradation rate. By altering the dosage, concentration, and duration of release, EFs present a platform to administer sustained therapy as a one-time application, reducing the need for patient adherence to multiple applications. As a first step toward this goal, we sought to evaluate the ability of PEO-based EFs loaded with *L. crispatus* to deliver viable probiotic bacteria, their ability to limit GBS colonization, and potential to modulate inflammatory changes in an *in vitro* vaginal epithelial cell model.

## RESULTS

### Electrospun fibers release viable *L. crispatus* that grow on vaginal epithelial cells

**Fig 1:**
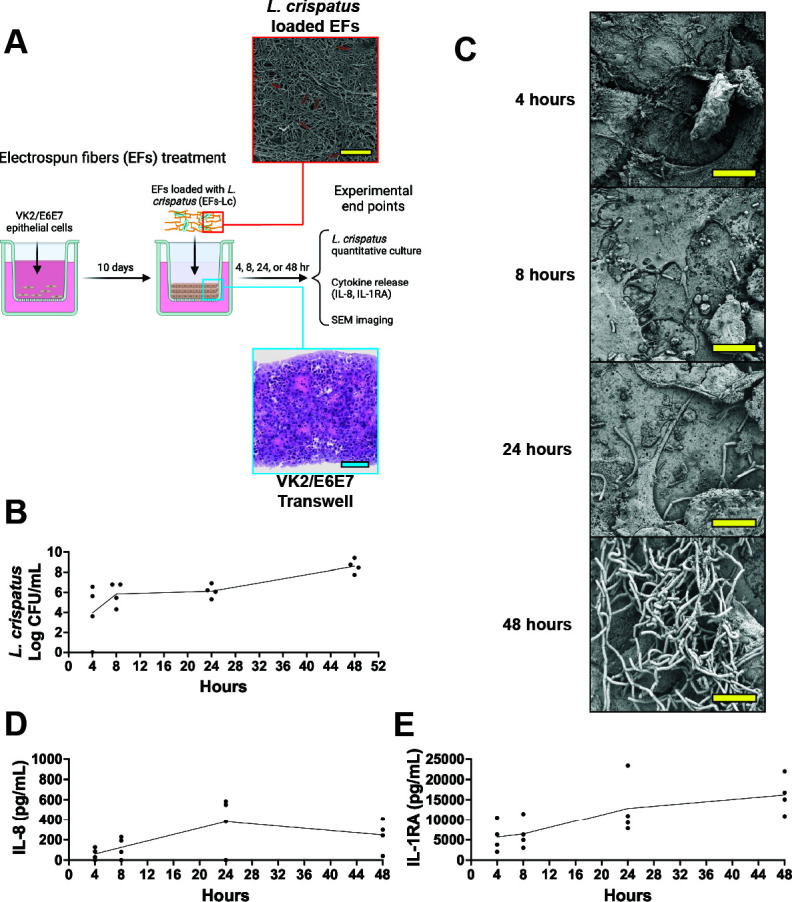
Electrospun fibers (EFs) release live *Lactobacillus crispatus* onto *in vitro* vaginal epithelial cells. (**A**) Schematic of experimental design describing how *L. crispatus-*loaded EFs were inoculated onto VK2/E6E7 transwells to complete the time course of *L. crispatus* release. Created in BioRender. Doster, R. (n.d.) https://BioRender.com/f46k967. Scanning electron microscopy (SEM) images of EFs loaded with *L. crispatus* (red box, *L. crispatus* cells are pseudocolored red). Also included, brightfield microscopy image of the histology transverse section of VK2 transwell stained with hematoxylin and eosin (blue box). (**B**) Quantitative culture of *L. crispatus* release from fibers over 48 hours. (**C**) SEM images of *L. crispatus* cells on vaginal epithelial cells over 48 hours. (**D, E**) Quantification of IL-8 (**D**) and IL-1RA (**E**) from basal supernatants from VK2 transwells over time. The blue measurement bar represents 100 μm, and the yellow bar represents 10 μm.

Our prior studies showed that EFs could be manufactured and incorporate viable *L. crispatus* (36–38). We sought to investigate *L. crispatus* release in the context of the vaginal epithelium in a controlled environment in which VK2/E6E7 vaginal epithelial cells are cultured on a semipermeable membrane at an air-liquid interface for an extended period to produce a multicellular epithelial structure *in vitro* (Fig. 1A). Electrospun fibers containing *L. crispatus* (EFs-Lc) were added to the upper chamber of VK/E6E7 transwells to evaluate the release of viable *L. crispatus* to the apical surface of vaginal epithelial cells over time. While the EFs visually dissolve within 2 hours (Fig. S1A), *L. crispatus* release and growth from the EFs increased over time and reached a mean of 8.29 × 10^8^ colony-forming units (CFU)/mL at 48 hours (Fig. 1B). These results were confirmed using scanning electron microscopy (SEM), and images demonstrated an increase in *L. crispatus* cells on the vaginal cell apical surface over 48 hours (Fig. 1C). To determine the effect of *L. crispatus* release on cytokine production by VK2/E6E7 transwells, we measured the proinflammatory cytokine interleukin 8 (IL-8) and anti-inflammatory IL-1 receptor antagonist (IL-1RA, also known as IL-1RN) release into the transwell lower chamber. These cytokines are among the most prominent pro- and anti-inflammatory cytokines produced by this model as multiplex cytokine analysis showed minimal production of other cytokines including IL-6, IL-10, TNF-α, IL-4, GM-CSF, MCP-1, IL-1β, IL-12p40, IFN- γ, IL-2, IL-12p70, IL-5, or IL-13 in response to GBS infection (Fig. S1B). IL-8 secretion peaked at 24 hours and then decreased at 48 hours (Fig. 1D), whereas that of IL-1RA increased over time and reached maximum release at 48 hours (Fig. 1E), mirroring the quantification of *L. crispatus* on the vaginal epithelial cells. Of note, while *L. crispatus* grew in the context of the VK2/E6E7 transwells, *L. crispatus* could not grow in vaginal cell culture media alone (Fig. S1C). These data suggest that the EFs incorporated viable *L. crispatus* cells that were released and grew in the context of the vaginal epithelium, leading to changes in cytokine signaling.

### EFs-Lc alter vaginal cell inflammatory signaling when added prior to GBS infection

**Fig 2:**
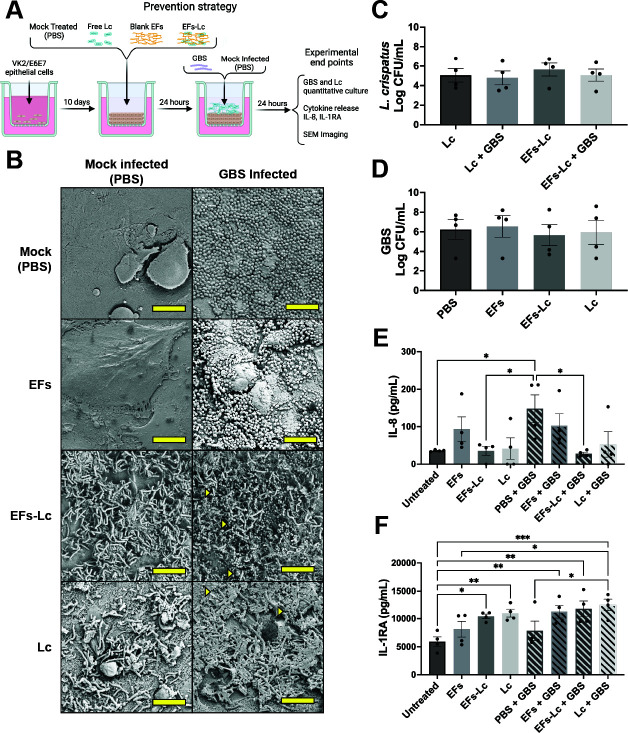
Effect of EFs-Lc application prior to GBS infection. (**A**) Schematic depicts the experimental design where VK2/E6E7 transwells were treated with EFs-Lc, blank EFs, free *L. crispatus* (Lc), or mock treatment (PBS) for 24 hours before GBS or mock (PBS) infection. Created in BioRender. Doster, R. (n.d.) https://BioRender.com/judciyo (**B**) SEM images of VK2/E6E7 transwells following treatments with EFs, EFs-Lc, *L. crispatus* (Lc), or mock treatment alone (left) or followed by GBS infection (right). Yellow measurement bar represents 10 μm, and yellow triangles denote areas of colocalization of GBS and *L. crispatus*. (**C, D**) Quantification of *L. crispatus* (**C**) and GBS (**D**) from the apical transwell chamber after treatment and 24 hours of GBS infection. (**E, F**) Quantification of IL-8 (**E**) and IL-1RA (**F**) from transwell basal supernatants. Bars represent the mean of four biological replicates ±SEM. Log-transformed bacterial quantification data were analyzed by Kruskal-Wallis with Dunns multiple comparison test and cytokine data were analyzed by one-way ANOVA with Tukey’s multiple comparison test. * denotes *P* < 0.05, ** denotes *P* < 0.01, and *** denotes *P* < 0.001. EFs: electrospun nanofibers; Lc: *L. crispatus*; PBS: phosphate-buffered saline.

To investigate the ability of EFs-Lc to mitigate or prevent GBS infection and manipulate the immune response of the vaginal epithelium, VK2/E6E7 transwells were treated with EFs-Lc, free *L. crispatus*, blank EFs, or mock treatment (phosphate-buffered saline, PBS) for 24 hours, followed by infection with GBS or mock infection (PBS) for another 24 hours (Fig. 2A). At the designated experimental end point, co-cultures were fixed and evaluated by SEM to verify the interactions between VK2/E6E7 cells, *L. crispatus*, and GBS (Fig. 2B). No fibers were seen by SEM at this time point. Images confirmed the release of *L. crispatus* from EFs and demonstrated colocalization of GBS and *L. crispatus* on the apical vaginal epithelial surface. Apical supernatants were collected for GBS and *L. crispatus* quantification to determine if pretreatment with EFs-Lc or *L. crispatus* affected GBS growth. *L. crispatus* was viable in the transwell model at 48 hours, but no significant difference was seen between groups, including from transwells infected with GBS (Fig. 2C). GBS quantitative culture showed no significant differences between the four experimental groups (Fig. 2D). We then investigated cytokine responses from the VK2/E6E7 transwells to examine if GBS, *L. crispatus*, or EFs altered vaginal cell inflammatory signaling. VK2/E6E7 transwells receiving mock treatment before GBS inoculation (PBS + GBS) produced the highest level of IL-8 release (147.9 ± 37.1 pg/mL); this difference was statistically significant when compared to untreated control, EFs-Lc and mock-infected, and EFs-Lc treated and GBS infected transwells (37 ± 1.2, 35.7 ± 12, and 28.5 ± 3.8 pg/mL, *P* < 0.05). EFs-Lc and *L. crispatus* pretreatment before GBS infection resulted in decreased IL-8 secretion to a level that was not significantly different from that of untreated controls (Fig. 2E). In contrast to IL-8, VK2/E6E7 transwells produced an abundance of the anti-inflammatory cytokine IL-1RA with the production significantly increased in transwells treated with *L. crispatus* (10964.2 ± 695.3 pg/mL, *P* ≤ 0.01), EFs-Lc (10396 ± 396.3 pg/mL, *P* ≤ 0.05), *L. crispatus* + GBS (12430.7 ± 1002.3 pg/mL, *P* ≤ 0.001), and EFs-Lc + GBS (11725.2 ± 1384.2 pg/mL, *P* ≤ 0.01) compared to the untreated control transwells (5924.1 ± 842.7 pg/mL) (Fig. 2F). Interestingly, the mean of transwells treated with EFs prior to GBS (EFs + GBS; 11213.5 ± 1128.7 pg/mL, *P* < 0.01) but not mock-infected transwells (EFs; 8123 ± 1387.4 pg/mL, *P* = 0.57) was significantly higher than the mean of the untreated controls. Altogether, these results highlight the pivotal role of *L. crispatus* in shifting inflammation signaling in the setting of GBS infection. IL-8 suppression was most pronounced when *L. crispatus* was delivered via EFs and *L. crispatus* delivered from culture or via EFs-Lc increased IL-1RA secretion compared to untreated controls. We also observed that blank EFs did not lead to a significant change in IL-8 or IL-1RA secretion compared to untreated transwells.

To understand if changes in VK2/E6E7 cell viability during the treatment and infections might be impacting cytokine secretion, we examined lactate dehydrogenase (LDH) release from the VK2/E6E7 transwells. When the apical supernatants were analyzed for LDH release as a marker of epithelial cell death, *L. crispatus-*treated, GBS-infected transwells contained significantly higher levels of LDH (1.34 ± 0.10, *P* < 0.05) similar to cells treated with Triton X-100 as a positive control (1.56 ± 0.11, *P* < 0.05) compared to untreated cells (0.40 ± 0.05) (Fig. S2). Literature suggests that *Lactobacillus* species secrete LDH (39), and when we assayed supernatants taken from *L. crispatus* cultures without VK2/E6E7 cells, we found high levels of LDH, confirming these reports (2.24 ± 0.12). GBS cultures showed negligible LDH release (0.08 ± 0.01). We observed no significant differences between the control and EFs-Lc treated samples, even though *L. crispatus* quantification was similar after treatment with EFs-Lc and free *L. crispatus* in the prevention model. We attribute this discrepancy to potential interference by polyethylene oxide (PEO) as it has been reported that polyethylene compounds can interfere with LDH activity (40, 41). In addition, there were no significant differences in LDH release compared to untreated control transwells in transwells treated with EFs, EFs-Lc, or infected with GBS.

### EFs and *L. crispatus* decrease GBS burden during *in vitro* established infections

**Fig 3:**
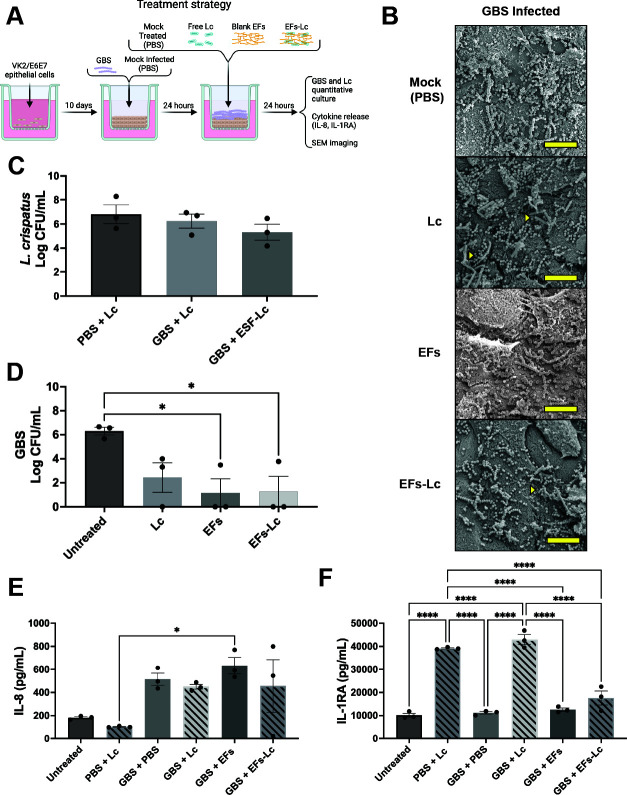
Effect of EFs-Lc application after GBS inoculation. (**A**) Schematic depicts the experimental design where GBS or mock infection was inoculated onto VK2/E6E7 transwells for 24 hours, followed by treatment with EFs-Lc, EFs, free *L. crispatus* (Lc), or mock treatment (PBS) for 24 hours. Created in BioRender. Doster, R. (n.d.) https://BioRender.com/ofok31r (**B**) SEM images of transwell apical surfaces following GBS infection and treatment. The yellow measurement bar represents 10 μm, and yellow triangles denote areas of colocalization of GBS and *L. crispatus*. (**C, D**) Quantification of *L. crispatus* (**C**) and GBS (**D**) from the apical transwell chamber after infection and 24 hours of the noted treatment. (**E, F**) Quantification of IL-8 (**E**) and IL-1RA (**F**) from transwell basal supernatants. Bars represent the mean of three biological replicates ±SEM. Log-transformed bacterial quantification data were analyzed by Kruskal-Wallis with Dunn’s multiple comparison test, and cytokine data were analyzed by one-way ANOVA with Tukey’s multiple comparison test. * Denotes *P* < 0.05, and **** denotes *P* < 0.0001. EFs: electrospun nanofibers; Lc: *L. crispatus*; PBS: phosphate-buffered saline.

While *L. crispatus* pretreatment with or without EFs prior to GBS infection altered vaginal cell inflammatory signaling, no change in GBS burden was noted (Fig. 2D through F). We then aimed to investigate the ability of EFs-Lc to disrupt preexisting GBS infection as a model of using EFs-Lc as a potential treatment. VK2/E6E7 transwells were infected with GBS or mock-infected (PBS) for 24 hours and then treated with EFs-Lc, EFs, free *L. crispatus*, or mock-treated (PBS) for 24 hours (Fig. 3A). SEM images showed the diffuse distribution of GBS on the apical surfaces of VK2/E6E7 transwells and colocalization between GBS and *L. crispatus* (Fig. 3B). *L. crispatus* quantitative culture showed no significant difference between *L. crispatus* treatment groups (Fig. 3C). Treatment of GBS-infected transwells with EFs-Lc and blank EFs significantly decreased GBS CFUs (2 × 10^3^ and 1 × 10^3^ CFU, *P* < 0.05, respectively) compared with untreated transwells (2.9 × 10^6^ CFU). There was no statistically significant difference in GBS CFU after treatment with *L. crispatus* compared to untreated samples (mean 4 × 10^3^, *P* = 0.07) (Fig. 3D). No statistically significant difference in IL-8 release was seen between GBS-infected transwells compared to the untreated cells (GBS + PBS 514.8 ± 52.5 pg/mL, *P* = 0.21, GBS + Lc 449.1 ± 20.7 pg/mL, *P* = 0.40; GBS + EFs 632.7 ± 70.8 pg/mL, *P* = 0.057; GBS + EFs-Lc 456.2 ± 228.4 pg/mL, *P* = 0.37 vs 182 ± 9.1 pg/mL). The only significant difference in IL-8 secretion among all conditions was seen in mock-infected transwells treated with free *L. crispatus* (PBS + Lc) compared with GBS-infected transwells treated with EFs (102.8 ± 3.5 pg/mL vs 632.7 ± 70.8 pg/mL, *P* ≤ 0.05) though there was no significant difference noted between GBS and mock-infected transwells receiving free *L. crispatus* (*P* = 0.18) (Fig. 3E). Transwells treated with free *L. crispatus* after mock or GBS infection secreted significantly higher amounts of IL-1RA than untreated controls or GBS-infected and mock-treated transwells (39,193.7 ± 285.6 pg/mL and 43,002.6 ± 2,141.4 pg/mL vs 10,147.8 ± 840.8 pg/mL and 11,166 ± 535.4 pg/mL, respectively, *P* < 0.0001). EFs or EFs-Lc treatment after GBS infection did not increase IL-1RA secretion compared to mock-treated transwells after 24 hours (Fig. 3F).

### Longer incubation with EFs-Lc after GBS infection promotes IL-1RA signaling

**Fig 4:**
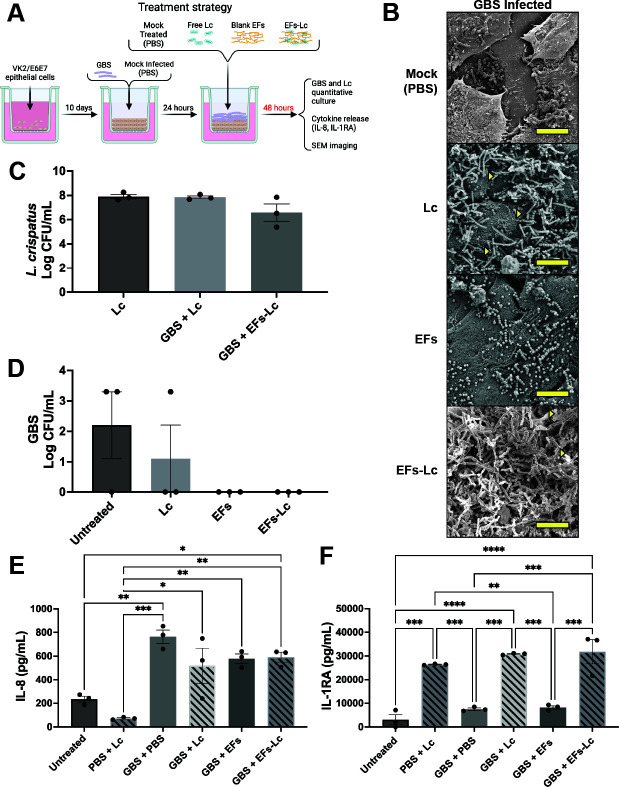
Effect of EFs-Lc application for 48 hours after GBS inoculation. (**A**) Schematic depicts the experimental design where GBS was inoculated onto VK2/E6E7 transwells for 24 hours followed by treatment with EFs-Lc, EFs, free *L. crispatus* (Lc), or mock treatment for 48 hours. Created in BioRender. Doster, R. (n.d.) https://BioRender.com/n48d970. (**B**) SEM images of transwell apical surfaces following infection and treatment. Yellow measurement bar represents 10 μm, and yellow triangles denote areas of colocalization of GBS and *L. crispatus*. (**C, D**) Quantification of *L. crispatus* (**C**) and GBS (**D**) from the apical transwell chamber after infection and 48 hours of the noted treatment. (**E, F**) Quantification of IL-8 (**E**) and IL-1RA (**F**) from transwell basal supernatants. Bars represent the mean of three biological replicates ±SEM. Log-transformed bacterial quantification data were analyzed by Kruskal-Wallis with Dunn’s multiple comparison test, and cytokine data were analyzed by one-way ANOVA with Tukey’s multiple comparison test. * Denotes *P* < 0.05, ** Denotes *P* < 0.01, *** denotes *P* < 0.001, and **** denotes *P* < 0.0001. EFs: electrospun nanofibers; Lc: *L. crispatus*; PBS: phosphate-buffered saline.

We sought to understand the lack of IL-1RA response from GBS-infected and EFs-Lc-treated transwells. Given that prior experiments on *L. crispatus* release and growth from fibers showed increasing numbers between 24 and 48 hours (Fig. 1B), we hypothesized that *L. crispatus* being released from the fibers may take longer to establish and become metabolically active before altering cytokine signaling. Consequentially, VK2/E6E7 transwells were infected with GBS for 24 hours and then treated with EFs-Lc, EFs, free *L. crispatus*, or mock treatment for 48 hours (Fig. 4A). SEM imaging confirmed pronounced growth of *L. crispatus* during the extra 24 hours (Fig. 4B). No significant differences in *L. crispatus* quantification were seen between the different treatment groups (Fig. 4C). Contrary to *L. crispatus*, the recovered GBS CFU decreased in untreated transwells infected with GBS, and no GBS was recovered from transwells treated with EFs or EFs-Lc, suggesting diminishing GBS survival in this model 72 hours after inoculation (Fig. 4D). Highest IL-8 secretion was observed in this transwell model at 48 hours after mock treatment in GBS-infected transwells (GBS + PBS) and was significantly higher than untreated control transwells and mock-infected transwells treated with *L. crispatus* (PBS + Lc) (763.4 ± 55.9 pg/mL vs 233.4 ± 25.5 pg/mL, 72 ± 6.2 pg/mL, *P* < 0.01 and 0.001, respectively). There was no statistically significant difference in IL-8 secretion of GBS-infected transwells treated with *L. crispatus*, EFs, or EFs-Lc compared to untreated, GBS-infected transwells (GBS + PBS). IL-8 secretion from GBS-infected transwells treated with *L. crispatus*, EFs, and EFs-Lc remained significantly higher than that of mock-infected, *L. crispatus*-treated (PBS + Lc) transwells (517.8 ± 148.8 pg/mL, 578.5 ± 40.2 pg/mL, 591.1 ± 42 pg/mL vs 72.1 ± 6.2 pg/mL, *P* < 0.05, 0.01, and 0.01, respectively). IL-8 secretion from GBS-infected transwells treated with EFs-Lc was significantly higher than that of untreated controls (591.1 ± 42 pg/mL vs 233.3 ± 25.5 pg/mL, *P* < 0.05) (Fig. 4E). Forty-eight hours of treatment with EFs-Lc after GBS infection (GBS + EFs-Lc) resulted in increased IL-1RA secretion compared with 24 hours of treatment (17,575.2 ± 3,034.1 pg/mL vs 29,148.8 ± 5,068.2 pg/mL, *P* < 0.05) (Fig. S3). Forty-eight hour IL-1RA release from GBS + EFs-Lc transwells was also significantly higher than that of the untreated control transwells, GBS-infected and -untreated transwells (GBS + PBS), and GBS-infected transwells treated with EFs (GBS + EFs) (29,148.8 ± 5,068.2 pg/mL vs 3,040.3 ± 2,110.2 pg/mL, *P* < 0.0001; 7,496.1 ± 507 pg/mL, and 8,226.8 ± 684.9 pg/mL, *P* < 0.001). Moreover, uninfected transwells treated with *L. crispatus* (PBS + Lc) had significantly higher IL-1RA secretion compared with untreated controls, GBS-infected and mock-treated (GBS + PBS), and GBS-infected transwells treated with blank EFs (GBS + EFs) (26,428.2 ± 157.7 pg/mL vs 3,040 ± 2,110.2 pg/mL, 7,496.1 ± 507 pg/mL, and 8,226.8 ± 684.9 pg/mL, *P* < 0.001, 0.001, and 0.01, respectively). Similarly, *L. crispatus* treatment of GBS-infected transwells (GBS + Lc) led to significantly higher IL-1RA levels at 48 hours compared with untreated, GBS-infected and mock-treated (GBS + PBS), and GBS-infected and EFs-treated transwells (GBS + EFs) (30,839.4 ± 234.8 pg/mL vs 3,040 ± 2,112.2 pg/mL, *P* < 0.0001; 7,496.1 ± 507 pg/mL, and 8,226.8 ± 684.9 pg/mL, *P* < 0.001) (Fig. 4F). Altogether, these results suggest that while GBS viability is limited in this model at 72 hours post-inoculation, *L. crispatus* growth eventually shifts inflammatory signaling toward an anti-inflammatory posture via IL-1RA release.

### GBS viability with EFs, EFs-Lc, and Lc in a simulated vaginal fluid

**Fig 5:**
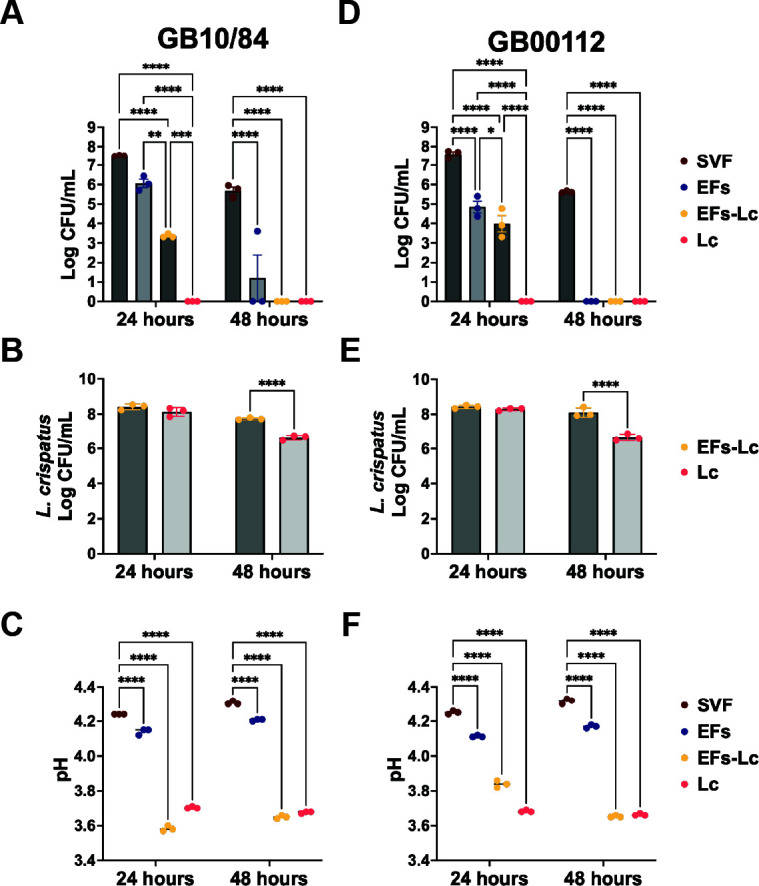
Impact of EFs, EFs-Lc, and *L. crispatus* on GBS survival in simulated vaginal fluid. GB10/84 (**A–C**) and GB00112 (**D–F**) were grown in SVF alone or with EFs, EFs-Lc, or Lc for 48 hours. (**A, D**) Quantification of GBS and *L. crispatus* (**B, E**) at 24 and 48 hours. (**C, F**) pH measurements at 24 and 48 hours. Data are presented as the mean ± SEM of three biological replicates and were analyzed by two-way ANOVA with Tukey’s multiple comparison test. * Denotes *P* < 0.05, ** denotes *P <* 0.01, *** denotes *P* < 0.001, and **** denotes *P* < 0.0001. SVF: simulated vaginal fluid; EFs: electrospun nanofibers; Lc: *L. crispatus.*

As our results showed that EFs and *L. crispatus* independently reduced GBS burden in the established GBS infection model. We investigated the contributions of anti-GBS activity by the different components using a simulated vaginal fluid (SVF) *in vitro* model. SVF supports the growth of both GBS and *L. crispatus* (Fig. S4). Strains GB10/84 and GB00112 were inoculated into SVF with EFs, EFs-Lc, free *L. crispatus*, or no treatment (control) and incubated for 48 hours (Fig. 5). GBS and *L. crispatus* were quantified at 24 and 48 hours, and pH was measured. At 24 hours, quantitative culture of GB10/84 showed a significant reduction following treatment with EFs-Lc (mean 2.3 × 10^3^ CFU, *P* < 0.0001) and free *L. crispatus* (no recoverable GBS detected, *P* < 0.0001) compared to SVF without treatment (mean 3.3 × 10^7^). Treatment with blank EFs resulted in significantly higher GBS CFU than those treated with EFs-Lc and *L. crispatus* (mean 1.4 × 10^6^ vs. 2.3 × 10^3^, and no recoverable GBS detected, *P* < 0.01, and 0.0001, respectively), while no significant difference was seen between SVF alone and cultures treated with blank EFs at 24 hours (*P* = 0.127) (Fig. 5A). GB00112 CFUs of samples treated with EFs, EFs-Lc, and *L. crispatus* were significantly lower than those of SVF without treatment (mean 1.1 × 10^5^, 2.2 × 10^4^, and no GBS detected, respectively, *P* < 0.0001, vs 4.3 × 10^7^). Similarly to GB10/84, the recovered GB112 treated with EFs was significantly higher compared to treatment with EFs-Lc or *L. crispatus* (mean 1.1 × 10^5^ vs 2.2 × 10^4^, and undetectable GBS, *P* < 0.05, and 0.0001, respectively) (Fig. 5D). At 48 hours, GB10/84 CFUs of untreated samples (mean 5.6 × 10^5^) were significantly higher than those of samples treated with EFs (mean 1.3 × 10^3^*, P* < 0.0001), EFs-Lc (no viable GBS recovered, *P* < 0.0001), and *L. crispatus* (no viable GBS recovered, *P* < 0.0001) (Fig. 5A). No GB00112 CFUs were recovered at 48 hours from samples treated with EFs, EFs-Lc, or *L. crispatus*, compared to untreated controls (mean 4.3 × 10^5^, *P* < 0.0001) (Fig. 5D). *L. crispatus* quantitative culture in the presence of GB10/84 showed no significant difference between EFs-Lc or *L. crispatus* treatments at 24 hours, while significantly more *L. crispatus* was viable at 48 hours in EFs-Lc samples than those treated with live *L. crispatus* from culture (mean 5.4 × 10^7^ vs 4.1 × 10^6^, *P* < 0.0001) (Fig. 5B). Similarly, in presence of GB00112, *L. crispatus* quantification showed a significant difference between samples treated with EFs-Lc and *L. crispatus* at 48 hours (mean 1.4 × 10^8^ vs 4.4 × 10^6^, *P* < 0.0001), while no difference was observed at 24 hours (Fig. 5E). As *L. crispatus* is known to acidify the vaginal environment, we examined how pH in this model may affect GBS viability. The initial pH, prior to GBS or *L. crispatus* inoculation, was 5.0, and was measured at 24 and 48 hours post-inoculation. At 24 hours, the pH of SVF cultures with GB10/84 alone decreased to 4.24 ± 0.00 but remained significantly higher than that of SVF treated with EFs (4.14 ± 0.01), EFs-Lc (3.58 ± 0.01), or *L. crispatus* (3.70 ± 0.003; for all treatments, *P* < 0.0001). Similarly, at 48 hours, the pH values were 4.30 ± 0.01 (untreated) vs 4.20 ± 0.003 (EFs), 3.65 ± 0.01 (EFs-Lc), and 3.67 ± 0.003 (*L. crispatus*) (for all treatments compared to SVF with GBS alone, *P* < 0.0001) (Fig. 5C). A similar pattern was observed for SVF cultures with GB00112. At 24 hours, the pH of SVF with GB00112 alone was 4.25 ± 0.005 compared to 4.11 ± 0.003 (EFs), 3.84 ± 0.011 (EFs-Lc), and 3.68 ± 0.003 (*L. crispatus*) (all, *P* < 0.0001). At 48 hours, pH values were similar at 4.31 ± 0.008 (untreated) vs 4.17 ± 0.005 (EFs), 3.65 ± 0.003 (EFs-Lc), and 3.66 ± 0.003 (*L. crispatus*) (all, *P* < 0.0001) (Fig. 5F). Altogether, the results demonstrate enhanced anti-GBS activity in the presence of *L. crispatus* at 24 hours in this model, likely in part through acidification of the environment, but EFs alone showed increasing anti-GBS activity over time by a pH-independent mechanism.

## DISCUSSION

Vaginal colonization with GBS is the major risk factor for ascending GBS infections during pregnancy. Previous studies have shown that the presence of *Lactobacillus* species is inversely correlated with GBS carriage (27, 42). Probiotic interventions to mitigate GBS carriage in pregnant women have been investigated as antibiotic-free approaches to reduce the need for IAP (43). As a first step toward more convenient probiotic delivery to prevent and treat infections, in this study, we sought to understand how EFs might be used to deliver *L. crispatus* in the context of vaginal epithelial cells and how *L. crispatus* might modulate colonization of GBS in a controlled environment. While EFs composed of PEO visually dissolved rapidly, *L. crispatus* release and growth were slower and increased after 24–48 hours, resulting in changes in vaginal cell inflammatory signaling that involved increases in IL-1RA and decreases in IL-8 secretion. Pretreatment of VK2/E6E7 transwells with EFs containing *L. crispatus* for 24 hours before GBS inoculation had minimal effect on the GBS burden in the model despite shifting the inflammatory balance through greater IL-1RA and decreased IL-8 secretion compared to GBS infection alone. When GBS infection of vaginal epithelial cells was established prior to treatment with EFs and *L. crispatus*, a decrease in GBS burden was observed in all groups including with EFs alone, suggesting that the fibers themselves impair GBS survival but do not affect *L. crispatus*. While 24 hours of treatment with EFs-Lc did not change IL-8 or IL-1RA release from GBS-infected transwells, after 48 hours of treatment with EFs-Lc, the IL-1RA response was restored, likely due to the longer period required for establishing *L. crispatus* colonization from the EFs compared to adding metabolically active *L. crispatus* from culture. We also evaluated *L. crispatus* and EFs anti-GBS activity in an *in vitro* SVF model. At 24 hours, both *L. crispatus-* and EFs-Lc treated cultures acidified the SVF, but only *L. crispatus-*treated cultures contained no viable GBS, whereas treatment with EFs-Lc or blanks EFs decreased GBS viability over time, suggesting pH-dependent and -independent mechanisms.

This model where VK2/E6E7 cells are cultured at an air-liquid interface (ALI) has benefits over traditional culture models using cell monolayers. ALI models have been used in numerous infectious and immunology studies and generate higher amounts of cytokines compared with monolayer culture and more faithfully mimic the structure, function, and the molecular features of the *in vivo* environment of the female reproductive tract (44–46). While this model represents an advance over monolayer cultures, it still represents a reductionist approach with limitations. While we used this model to examine interactions between host cells, commensal bacteria, and opportunistic pathogens, the remainder of the vaginal microbiota is missing, which does not currently allow for complex microbe-microbe interactions. Close interactions between *L. crispatus* and the VK2/E6E7 cells can be evaluated as *L. crispatus* is not able to grow in the cell culture media but grows well in the context of the vaginal epithelial cells. GBS grows on the apical surface of these cells, including in biofilm structures, and these biofilms are thought to contribute to vaginal colonization (47). Another limitation of the current model is the inability to reliably measure the pH, lactic acid, or hydrogen peroxide on the apical cell surface. Small volumes were used to deliver bacteria to the surface while maintaining the air-liquid interface of the cultures, preventing consistent measurement of changes that lactobacilli are thought to promote to limit colonization by potential pathogens. GBS survival in this model was found to decrease 72 hours post-inoculation. VK2/E6E7 cells have been shown to produce several antimicrobial proteins including SLPI, human beta defensins, and S100A7 (48). Other studies examining pathogen-VK2/E6E7 interactions have noted increased production of these proteins in response to pathogens such as *Gardnerella* (49), which may explain the decreased GBS survival over time. Lastly, the layered, multicellular structure of the model and using multiple microorganisms present unique challenges in assessing changes in vaginal cell viability or number during the models, which may affect cytokine secretion. We have previously shown that EFs and/or *L. crispatus* exposure does not impact VK2/E6E7 cell viability (38), but live bacteria can affect the readout of several different cell viability assays including LDH (50), MTT (51), and ATP assays (52). LDH readings from the apical chamber demonstrated higher LDH signal from transwells that received free *L. crispatus*, and we subsequently found that *L. crispatus*, but not GBS, grown in culture secrete LDH, as has been reported for other *Lactobacillus* species (39). Interestingly, we did not see the same increase from EFs-Lc treated samples, and prior studies have suggested polyethylene compounds can interfere with LDH activity by forming unproductive ternary complexes of NAD-pyruvate-LDH (40, 41). VK2/E6E7 viability may be masked or underestimated in these experimental conditions, which may impact the variability of cytokine secretion seen between the models.

The SEM images demonstrate close interactions between GBS and *L. crispatus* on the vaginal cell surface. Various *in vitro* studies have investigated the antagonistic activity of *Lactobacillus* species against GBS. This activity is attributed to various mechanisms including niche acidification through lactic acid production, secretion of bacteriocins and hydrogen peroxide, coaggregation with the pathogen, adhesion to epithelial cells, and modulation of immune signals (53–55). Past studies have demonstrated decreasing viability when GBS was exposed to pH below 5 or lactic acid in a modulated environment (56–58). We sought to understand some of these changes using a second model, in which SVF was used to compare the anti-GBS activity *of L. crispatus* and blank EFs. *L. crispatus* demonstrated superior anti-GBS activity compared to blank EFs in SVF. No GBS was detected after treatment with free *L. crispatus* in SVF, and EFs-Lc significantly reduced GBS CFU compared to blank EFs at 24 hours despite resulting in similar decreases in GBS CFU in the VK2/E6E7 transwell models (Fig. 3D and 4D). All treatments led to decreased viability of GBS in SVF over 48 hours. Part of the anti-GBS activity appears to be related to *L. crispatus* acidification of the environment as pH measurements were significantly lower in cultures treated with live *L. crispatus* or EFs-Lc. While the pH measurements at 24 hours were similar between those treated with free *L. crispatus* compared to EFs-Lc, viable GBS was obtained at 24 hours from cultures treated with EFs-Lc but not free *L. crispatus*. Whether these differences are related to a more rapid growth and acidification from free *L. crispatus* cells over those being released from the EFs or a pH-independent mechanism of GBS killing is not clear from these studies.

We did see several differences between our models treated with EFs-Lc and free *L. crispatus* from culture in these studies as treatment with free *L. crispatus* led to more robust IL-1RA release after 24 hours of treatment than that from EFs-Lc and more rapid GBS killing in SVF. While we recovered similar numbers of *L. crispatus* cells between the groups, the data suggest the *L. crispatus* cells are not functioning equally in the models. Initial *L. crispatus* quantification within the transwells (Fig. 1B) suggests that despite rapid dissolving of the PEO EFs, *L. crispatus* growth in the VK2/E6E7 transwell model may not occur until near 24 hours as we see quantification surpassing the initial inoculum after 24 hours. It may be that the difference in growth and metabolism of *L. crispatus* from the EFs-Lc is delayed compared to those from culture, leading to slower acidification and subsequent immunomodulation. We also observed differences in mean *L. crispatus* quantification between the models, and whether this variability is the result of the complexities of the model including prolonged epithelial culture and coordinating delivery of two different microorganisms or other factors will require additional investigation, but this variability may reduce the statistical power to detect some differences between treatment groups. Future studies will need to examine these differences to better understand the impact of the release of *L. crispatus* within the vaginal niche.

In this model, *L. crispatus*-independent killing was observed after GBS infection was established as EFs alone resulted in a significant decrease in GBS burden. Antimicrobial mechanisms of polymers including PEO are not well defined. Studies suggest that the acidic nature of polymers such as PEO may augment antimicrobial activity by acting on the bacterial surface via charge directly or binding metals that stabilize the cell surface (59, 60). Few studies have examined the antimicrobial activity of PEO directly, but many rather focus on using PEO to deliver compounds with known antibacterial properties including antibiotics, silver nanoparticles, or chitosan (59, 61). For instance, a study examining the use of PEO electrospun fibers to deliver soy protein as a wound dressing determined the co-formulated electrospun webs had antimicrobial activity against *Staphylococcus aureus* and *Pseudomonas aeruginosa*, but the PEO fibers alone had no activity (62). A similar study investigated the use of chitosan (CS)-PEO nanofibrous mats containing different amounts of silver nanoparticles and compared the antibacterial activity against *S. aureus* and *Escherichia coli*. CS-PEO mats alone showed some activity against the gram-positive *S. aureus* but not against *E. coli,* and the authors suggest that the outer bacterial membrane of gram-negative species may provide resistance to the activity of polymers (63). Direct comparisons with these studies are difficult given differences in electrospun fiber formulation, concentration, duration of treatment, and methods for measuring antimicrobial activity. Our SVF studies suggest EFs treatment leads to mild acidification of the environment, but future studies will investigate the mechanism by which PEO EFs may have selective inhibition to GBS and other pathogens, while encapsulated *L. crispatus* appeared to grow without impairment.

When evaluating the inflammatory responses of this model, we focused on two cytokines with different functions, IL-8 and IL-1RA. Multiplex cytokine analysis showed that these cells produce very little TNF-α, IL-1β, IL-4, IL-10, IFN-γ, IL-2, or MCP-1 in response to GBS infection, and similar findings have been documented by others (64, 65). IL-8 is a pro-inflammatory chemokine important for neutrophil trafficking, and IL-8 release by VK2/E6E7 cells has been shown to model mucosal toxicity in animal models of vaginal inflammation (66). VK2/E6E7 cells release IL-8 in response to a variety of vaginal pathogens including GBS via stimulation of CD40 and TLR-2 (67). IL-1RA blocks the inflammatory activity of IL-1 by binding to the IL-1 receptor. Numerous studies have demonstrated links between lactobacilli and production of vaginal cell IL-1RA (4, 68, 69). Lactic acid produced by *Lactobacillus* species is thought to inhibit activation of NF-κB pathways and contribute to immunomodulatory activity within the vagina (65). The influence of the microbiome on vaginal inflammation is well documented. Vaginal IL-8 and other inflammatory cytokines are inversely associated with the presence of *Lactobacillus* species (70, 71), which was further confirmed in our models. Vaginal inflammation has heightened consequences during pregnancy, where reduced vaginal lactobacilli, and thus, IL-1RA levels, in the cervicovaginal fluid of pregnant women are linked to spontaneous preterm delivery (72), highlighting the potential benefits of using probiotics during pregnancy. Congruent with these studies, our results demonstrate that pretreatment with EFs-Lc before GBS infection modulated the immune signals by decreasing IL-8 cytokine levels and increasing IL-1RA but did not affect GBS burden. The treatment model using EFs-Lc after GBS establishment resulted in decreased GBS burden and elicited a similar immune signaling shift toward IL-1RA. We saw varying amounts of IL-1RA released from the GBS prevention and treatment models. Further studies are needed to understand if the sequence of microbial signals provided to the VK2/E6E7 cells leads to changes in IL-1RA secretion or if enhanced IL-1RA release acts as a counterbalance to enhanced IL-8 secretion seen in the treatment model where GBS infection is established before EFs application.

EFs have been considered a promising platform for delivering probiotics intravaginally. The high surface area-to-volume ratio provides a high load of *L. crispatus,* and the biocompatible matrix made of PEO preserves bacterial viability and morphology. The results indicated that the EFs release *L. crispatus* cells that remain viable and grow on the vaginal epithelium. *L. crispatus* morphology and structure integrity were confirmed by SEM imaging. Several clinical trials have tested probiotics to treat GBS-colonized pregnant women in late gestation without reporting adverse events but have shown mixed outcomes in terms of the efficacy to decrease GBS colonization. The outcomes depended on several parameters including the type of probiotic, the length of the interventions, the dose, and whether a single probiotic or a combination was used (28, 43, 73, 74). For example, one clinical trial consisted of daily oral supplements of *L. rhamnosus* GR-1 and *L. reuteri* RC-14 to decrease vaginal and rectal GBS colonization in pregnant patients; 43% of GBS-positive pregnant women achieved negative GBS culture results over approximately 3 weeks (28). Meta-analysis of this and similar studies suggests that probiotic interventions could reduce GBS colonization, but further evaluation is needed to maximize *Lactobacillus* strain choice, dose, and route of administration (43). For this purpose, analyses leveraging computational modeling and simulation may help explore the parameter space and identify values to maximize the efficacy, as has been shown previously (75–79). Intravaginal delivery of probiotics has the benefit of not requiring passage through the acidic stomach, which could lead to loss of probiotic bacteria. Fewer studies have examined vaginal administration of probiotics to combat GBS colonization, but vaginal probiotics have shown promise for other conditions including bacterial vaginosis and urinary tract infections (80, 81). Probiotic use may be of particular interest in populations at increased risk for GBS colonization, such as those with pregestational diabetes who are at least twice as likely to have GBS colonization when their hemoglobin A_1c_ is greater than 6.5% (82, 83).

As maternal health progresses toward individualized treatments, electrospinning offers the potential to devise versatile active pharmaceutical ingredient formulations tailored to individual needs. One of the benefits of EFs is that manufacturing modifications could be made to improve success of probiotic delivery. Use of different polymers and 3D printing structures provides opportunities for variable ranges of fiber dissolution and encapsulant release. While this study used rapidly dissolving fibers due to the relatively short time span of the model, prior work has demonstrated formulations with sustained release for over 2 weeks, which may be more appropriate for clinical applications (36). EFs synthesis also provides opportunities to use different *Lactobacillus* species and incorporate other components such as antibiotics targeting vaginal anaerobes, like metronidazole, which may help shift the vaginal microbiome and better support lactobacilli growth to establish prolonged colonization (37). Importantly, this study shows the fibers do not appear to be irritating to the vaginal epithelium, consistent with findings of previous work showing no significant inflammation when EFs were used in a preclinical model using estrogenized mice (37).

Further studies are needed to optimize the effect of EFs-Lc. As stated above, several modifications can be explored, including use of different *Lactobacillus* species, co-delivery with antibiotics, and modifying fiber dissolution rates to impact the timing and duration of delivery. These therapy parameters could be explored with computational modeling. Additionally, further investigation is needed regarding the impact of vaginal inflammatory signaling of cytokines such as IL-8 and IL-1RA on responding cells such as neutrophils during vaginitis (84). Future studies will further investigate the impact of the fibers on pathogenic and commensal bacteria as the mechanisms for the observed anti-GBS activity are unclear. These studies will culminate in preclinical models to evaluate the effectiveness of EFs-Lc to improve female reproductive health in the face of diverse infections.

In summary, this study shows the potential of EFs to deliver viable commensal bacteria to the vaginal mucosa infected with GBS. While prior treatment with EFs-Lc did not prevent GBS establishment on vaginal transwells, treatment with EFs-Lc did alter the inflammatory signaling toward IL-1RA production. EFs alone or with *L. crispatus* decreased GBS survival 24 hours post-treatment. This study underscores the potential of localized delivery alternatives for GBS infection to reduce complications such as preterm labor and neonatal sepsis. Future work will investigate ways to maximize *L. crispatus* delivery and the establishment of a potential therapeutic solution that stabilizes a *Lactobacillus* dominant vaginal microbiome and decreases GBS colonization during pregnancy.

## MATERIALS AND METHODS

### Synthesis of electrospun fibers (EFs)

Polyethylene oxide (PEO) (600,000 MW) was purchased from Sigma Aldrich (St. Louis, MO). De Man, Rogosa, and Sharpe (MRS) was purchased from Sigma Aldrich (St. Louis, MO) for dissolution of PEO for electrospinning fiber solutions.

Fibers with “rapid release” capabilities, characterized by their full dissolution in less than 24 hours, were constructed utilizing 250 mg PEO (5% wt/vol) dissolved in 5 mL of MRS broth. For *L. crispatus*-loaded PEO fibers, 5 × 10^7^ CFU *L. crispatus*/mg PEO solution was centrifuged (3,500 × g, 10 min), the supernatant was removed, and the *L. crispatus* pellet was resuspended with 500 µL of MRS. Then, the *L. crispatus* solution was added to 4.5 mL of MRS broth that contained 5% wt/wt PEO. To minimize the risk of fiber contamination, polymer solutions were passed through a 0.45 µm syringe filter (VWR, Radnor, PA) before probiotic incorporation. Additionally, the electrospinning box and all materials in contact with fibers were sterilized prior to electrospinning. During electrospinning, a positive voltage of 25 kV was applied at the tip of a needle connected to a syringe containing PEO. EFs were collected on a rotating mandrel, positioned 15 cm from the needle tip. The syringe flow rate was 0.3 mL/hour for EFs. Electrospinning conditions were maintained at 35% humidity with room temperature.

### Bacterial culture

*Streptococcus agalactiae* strain CNCTC 10/84 (ATCC, number 49447, denoted as GB10/84 in this study) and strain GB00112, representing capsular type V and III, respectively, were isolated from a case of neonatal sepsis and rectovaginal swab from woman persistently colonized before and after childbirth (85, 86). GB10/84 was chosen for these studies as it has been used extensively in *in vitro* human vaginal and *in vivo* mouse colonization models (87, 88) and has shown significant adherence to VK2/E6E7 cells, the vaginal epithelial cell line used in this study (88). Bacterial cells were cultured on tryptic soy agar plates supplemented with 5% sheep blood (blood agar plates) at 37°C overnight. Bacteria were subcultured from blood agar plates into THB and incubated under shaking conditions at 37°C in ambient air to the stationary phase. Cultures were centrifuged to pellet bacterial cells and were then resuspended in sterile PBS. Cultures were then measured with a spectrophotometer to determine the bacterial density at an optical density of 600 nm (OD_600_) to calculate the volume needed to deliver 10^7^ CFU. Quantitative culture was conducted by obtaining samples at noted time points that were serially diluted in sterile PBS and plated on blood agar plates.

For probiotic loading in the electrospinning solution, *L. crispatus* (strain MV-1A-US, BEI Resources number HM-637) was cultured on MRS (supplemented with 0.1% Tween 80) agar plates in a closed, anaerobic system with generated carbon dioxide at 37°C for 48 hours. When colony formation was visually evident on the agar plates*, L. crispatus* was subcultured by selecting a single colony and culturing in MRS broth at 37°C for an additional 24 hours in the same conditions. Cultures were measured with a spectrophotometer to determine the volume of the *L. crispatus* solution to obtain a theoretical loading of 5 × 10^7^ CFU.

### *In vitro* vaginal epithelial cell culture model

Human vaginal epithelial VK2/E6E7 cells (ATCC CRL-2616) were cultured in keratinocyte-serum-free medium (KSFM, Gibco, Waltham, MA) supplemented with 0.1 ng/mL human recombinant EGF, 0.05 mg/mL bovine pituitary extract, 44.1 mg/L calcium chloride, and PEN-STREP antibiotic-antimycotic mixture (Gibco). Cells were subcultured using 0.25% trypsin-EDTA (1X) and 1:1 mixture of Dulbecco’s modified Eagle’s medium and Ham’s F12 medium (DMEM: F-12, Gibco) supplemented with 10% fetal bovine serum (Gibco). Cultures were incubated at 37°C in 5% CO_2_. Then, 12 mm transwell permeable membranes (0.4 μm pore size, Corning 3460) were seeded with VK2/E6E7 cells (60,000 cells/well) in the upper chamber, and 1 mL KSFM was added to the lower chamber, as previously described (44). For 10 consecutive days, the medium was aspirated from the upper/apical chamber and that in the lower/basal chamber was replaced with fresh media to establish the air-liquid interface. An antibiotic-free culture medium was used starting 24 hours prior to infection. Lower chamber cell culture medium was changed daily during infection/treatment experiments.

To model using EFs to prevent GBS colonization (prevention model), transwells were pretreated with either 5 × 10^7^ CFU of free *L. crispatus*, EFs containing 5 × 10^7^ CFU *L. crispatus* (EFs-Lc), or blank EFs without lactobacilli (EFs). Transwell cultures were maintained at 37°C in 5% CO_2_ for 24 hours. After 24 hours, lower compartment cell culture medium was changed, and transwells were infected with GB10/84 cells (10^7^ per transwell) in PBS. PBS alone was used for mock-infected transwells. GBS or mock-infected transwells were incubated for another 24 hours at 37°C in 5% CO_2_. To evaluate whether EFs with or without *L. crispatus* could disrupt GBS colonization (treatment model), VK2/E6E7-seeded transwells were infected with GB10/84 (10^7^ CFU per transwell) or mock (PBS) for 24 hours, followed by treatment with free *L. crispatus*, EFs-Lc, or EFs without lactobacilli for 24 or 48 hours. At the designated endpoint, basal and apical supernatants were collected for cytokine analysis (basal) and quantitative culture (apical). Co-culture membranes were fixed for scanning electron microscopy imaging.

### Field-emission scanning electron microscopy

After VK2/E6E7 transwells were treated as described above, transwell membranes were carefully extracted and incubated in 2.0% paraformaldehyde and 2.5% glutaraldehyde in 0.05 M sodium cacodylate buffer for at least 24 hours prior to sequential dehydration with increasing concentrations of ethanol. Transwells were dried at the critical point, using a Samdri-795 critical point CO_2_ dryer (Tousimis, Rockville, MD), mounted onto an aluminum stub, and sputter-coated with 80/20 gold-palladium using a Cressington 108 auto Sputter Coater (Cressington Scientific Instruments, Watford, United Kingdom). A thin strip of colloidal silver (Electron Microscopy Sciences, product 12630, Hatfield, PA) was painted at the sample edge to dissipate sample charging. Transwells were imaged with a ThermoFisher Apreo C LoVac Field Emission Scanning Electron Microscope.

### Quantification of cytokines

Supernatants from the basal chamber of transwells were evaluated for release of cytokines including human IL-8/CXCL8 and IL-1RA/IL-1F3 using DuoSet ELISA kits (R&D System, Minneapolis, MN) according to the manufacturer’s protocol, and protein levels were calculated from the standard curves. Multiplex cytokine analysis was conducted on basal supernatants from VK2/E6E7 transwells infected with GBS for 24 hours. Human Focused 15 Plex Discovery Assay was conducted by Eve Technologies (Calgary, AB Canada).

### Bacterial growth analysis

To examine *L. crispatus* and GBS growth in different media, bacterial cells were subcultured from overnight cultures as above and inoculated from the stationary phase into wells of a polystyrene plate containing either KSFM without antibiotics, MRS broth (*L. crispatus*), THB (GBS), or simulated vaginal fluid. Cultures were incubated in a Cerillo Alto Optical Density Microplate Reader (Cerillo, Charlottesville VA) at 37°C in ambient air, with OD_600_ readings taken every 15 minutes.

### Lactate dehydrogenase (LDH) cell viability assay

To assess how different treatments might affect the vaginal cell viability, the prevention modeling of EFs-Lc treatment prior to GBS infection was performed as described above, but at the designated end point, 10 μL of the VK2/E6E7 transwell apical supernatants, supernatant from *L. crispatus* grown in MRS for 48 hours, or GBS grown in THB for 48 hours were diluted with KSFM cell culture media (1:5), and lactate dehydrogenase release was quantified using the CyQuant LDH assay kit (Invitrogen) according to the manufacturer’s protocol. As the *L. crispatus* and GBS cultures were grown separately, results are shown as OD_490_ values from the assay rather than a percent of maximal VK2 LDH release.

### Simulated vaginal fluid GBS and *L. crispatus* cultures

Simulated vaginal fluid (SVF) was prepared as previously described (89) with the following modifications to support GBS growth: glucose and sodium acetate were omitted, and the concentration of lactic acid was reduced to achieve a starting pH of 5.0. GBS strains GB10/84 and GB00112 were cultured overnight in THB, then subcultured into fresh media, and grown to the mid-log phase. Cultures were centrifuged to pellet the bacteria, which were then resuspended in sterile PBS. A total of 10⁷ CFU of GBS were added to 1 mL of SVF with or without EFs, EF-Lc, or free *L. crispatus* (5 × 10^7^ in 100 μL MRS). Co-cultures were incubated for 48 hours at 37°C in 5% CO_2_. At 24 and 48 hours, GBS and *L. crispatus* CFU were quantified, and pH was measured using a SevenDirect SD20 pH meter (Mettler Toledo).

### Statistical analysis

All *in vitro* experiments were performed at least three independent times, and results were recorded as the mean ± standard error. Comparison of two groups (multiplex cytokine data) was completed using a two-tailed *t*-test. Comparison of three or more groups was completed using a one-way ANOVA with correction for multiple comparisons using *post hoc* Tukey test for normally distributed data (cytokine analysis and LDH assay) and Kruskal-Wallis test with *post hoc* Dunn’s test for nonparametric data (log-transformed bacterial quantification). Changes in GBS and *L. crispatus* CFU over time and pH studies with SVF were analyzed by two-way ANOVA with correction for multiple comparisons using Bonferroni test. Statistical analyses were performed using GraphPad Prism version 10.3.0. *P* < 0.05 was considered statistically significant.

## Data Availability

Data will be made available upon reasonable request.

## SUPPLEMENTAL MATERIAL

Supplemental figures iai.00170-25-s0001.pdf Figures S1 to S4.

ASM does not own the copyrights to Supplemental Material that may be linked to, or accessed through, an article. The authors have granted ASM a non-exclusive, world-wide license to publish the Supplemental Material files. Please contact the corresponding author directly for reuse.
