# Supplementary material for: Modulation of group B Streptococcus infection and vaginal cell inflammatory signaling in vitro by Lactobacillus crispatus-loaded electrospun fibers
Source: Infect Immun. 2025 Aug 27;93(10):e00170-25. doi: 10.1128/iai.00170-25 (PMC12519788; doi:10.1128/iai.00170-25)
Supplement: Supplemental figures — Figures S1 to S4. [file iai.00170-25-s0001.pdf]

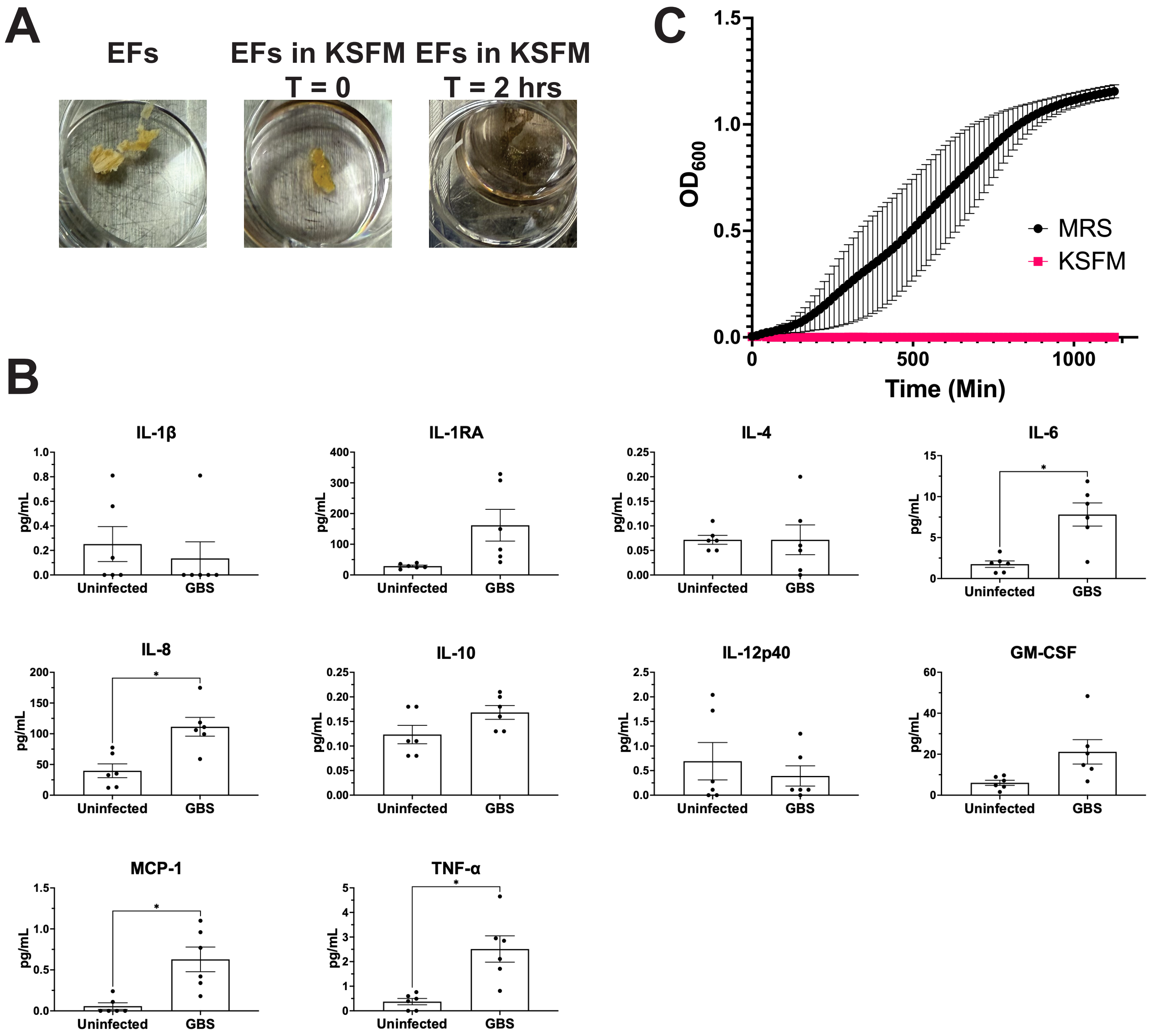

Supplemental Figure 1: Host-pathogen interactions in the VK2/E6E7 transwell model. (A) Image of EFs and dissolution in KSFM at 0 and 2 hours. (B) Multiplex cytokine quantification of VK2/E6E7 transwells infected with GBS for 24 hours. Bars represent the mean of six biological replicates ± SEM. IFN- γ, IL-2, IL-12p70, IL-5, or IL-13 were measured but were below the assays limit of detection and are not shown. Data represent the mean ± SEM of six biological replicates and were analyzed by paired, two-tailed t test. * Denotes $p < 0.05$. (C) Growth of *L. crispatus* strain MV-1A-US in MRS and KSFM. Data represent the mean ± SEM of three biological replicates. EFs: electrospun nanofibers; KFSM: keratinocyte-serum-free medium; MRS: De Man, Rogosa, and Sharpe.

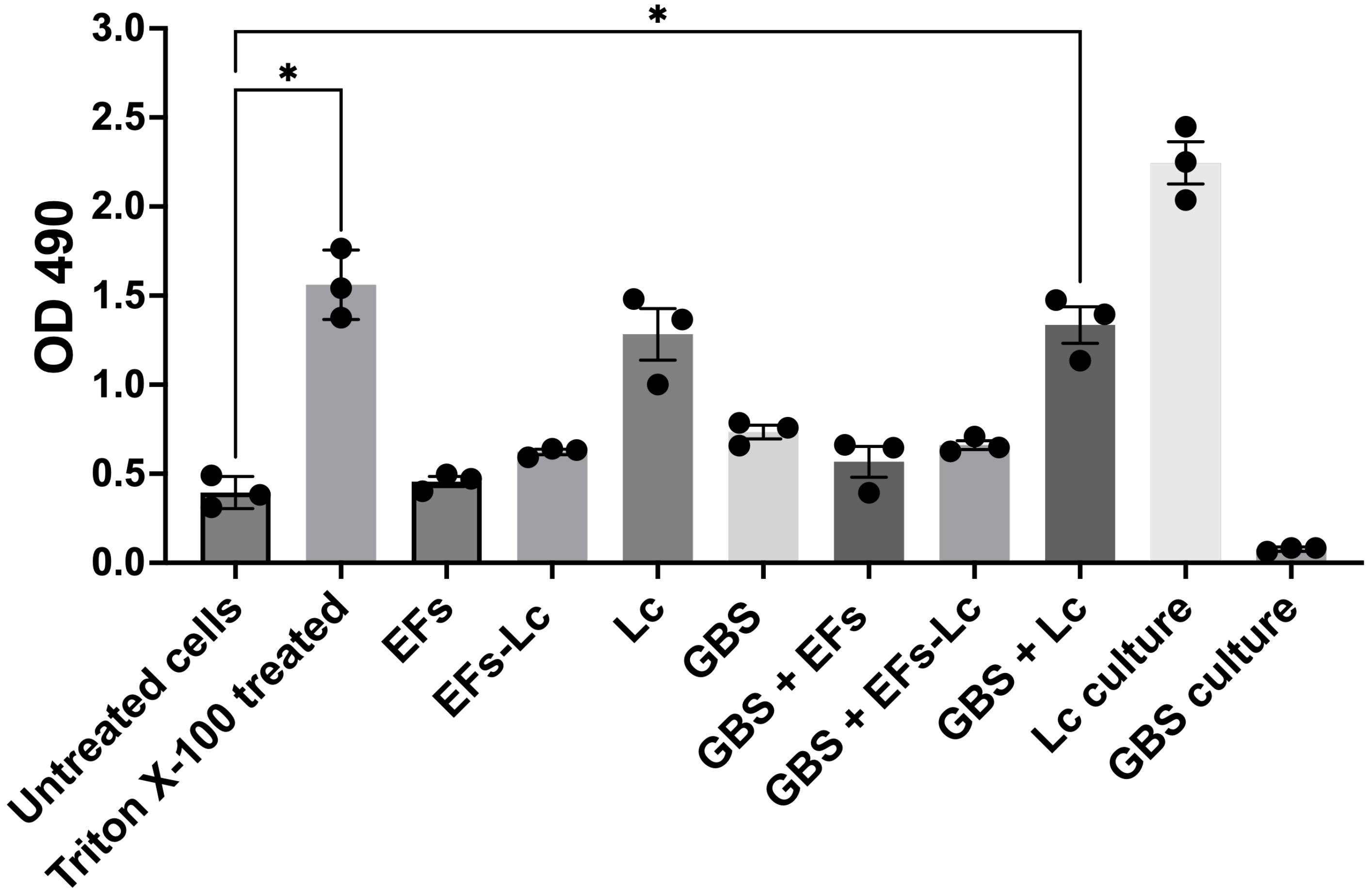

Supplemental Figure 2: Cell viability of VK2/E6E7 transwells. Transwells were treated as described in Figure 2A. Apical supernatants from transwells, supernatant from *L. crispatus* cultured for 48 hours in MRS, or GBS cultured for 48 hours in THB were sampled and assayed for LDH release. Data are shown as the mean OD_{490} readings ± SEM of three biological replicates and were analyzed by one-way ANOVA with Dunnett multiple comparison test with groups compared to untreated cell control. *L. crispatus* and GBS culture supernatants were excluded from statistical comparison. * denotes $p < 0.05$. EFs: electrospun fibers; Lc: *L. crispatus.*

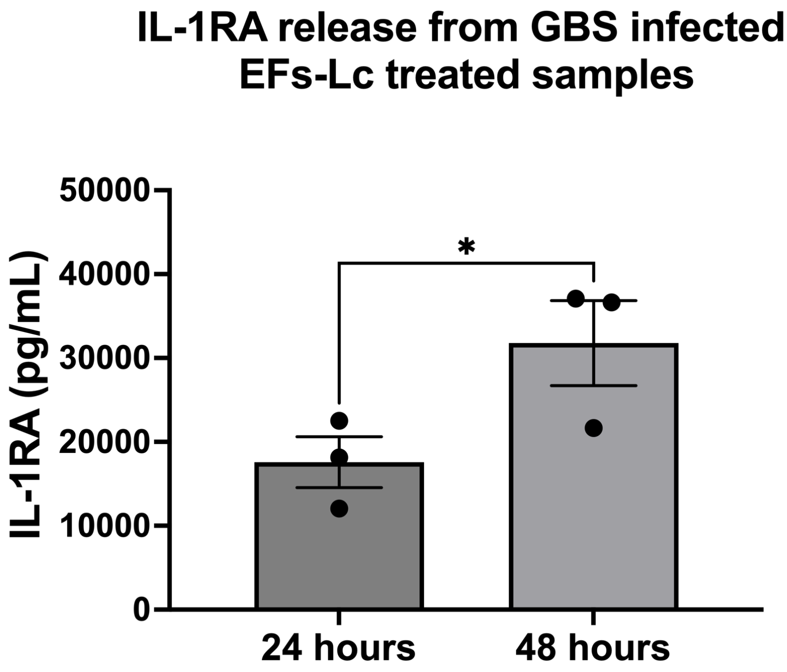

Supplemental Figure 3: IL-1RA release in GBS infected, EFs-Lc treated transwells at 24 and 48 hours. Comparison of IL-1RA release from GBS infected, EFs-Lc treated transwells after 24 hours or 48 hours of treatment (Figs. 3F and 4F). Data analyzed by unpaired, one-tailed t test. * Denotes $p < 0.05$.

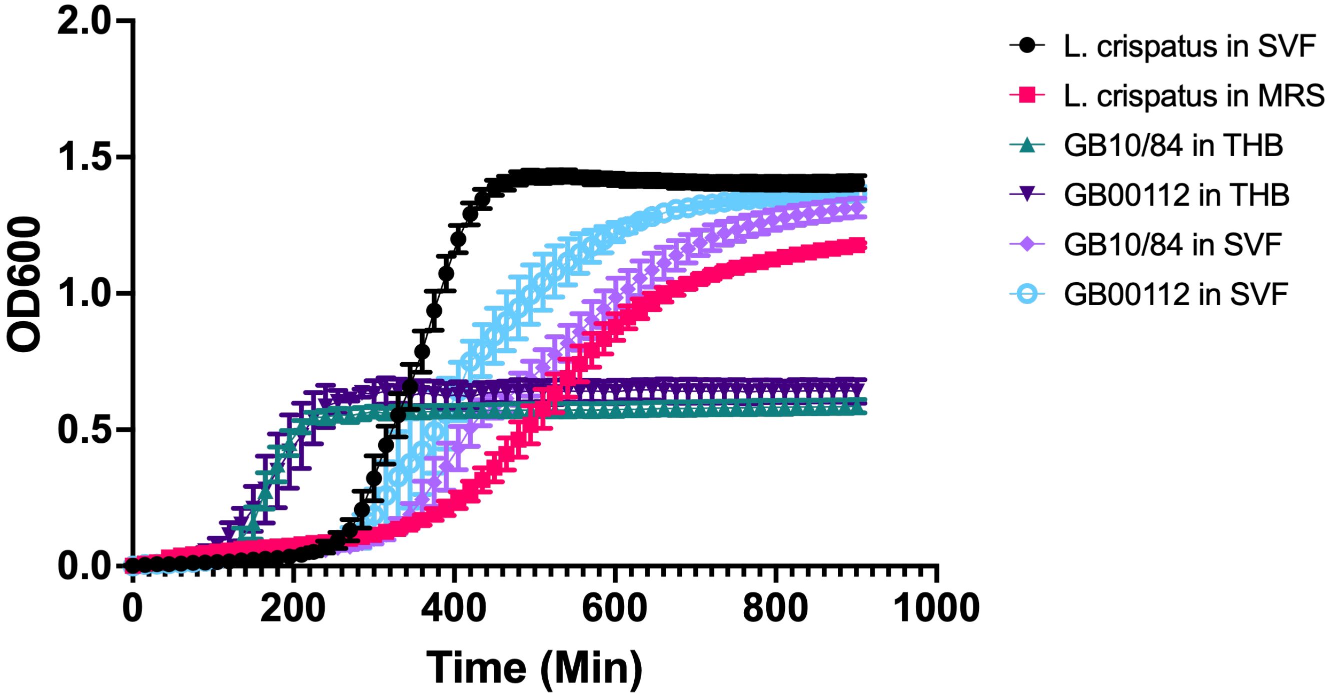

Supplemental Figure 4: Growth of *L. crispatus* and GBS strains GB10/84 and GB00112 in SVF. Bacterial cells were grown in SVF, MRS (*L. crispatus*), or THB (GBS) with OD_{600} readings taken ever 15 minutes to evaluate bacterial growth. Data represent the mean ± SEM of three biological replicates. SVF: simulated vaginal fluid; THB: Todd Hewitt broth, MRS: De Man, Rogosa, and Sharpe broth.
